# Implementation of Evidence-based Asthma Interventions in Post-Katrina New Orleans: The Head-off Environmental Asthma in Louisiana (HEAL) Study

**DOI:** 10.1289/ehp.1104242

**Published:** 2012-08-15

**Authors:** Herman Mitchell, Richard D. Cohn, Jeremy Wildfire, Eleanor Thornton, Suzanne Kennedy, Jane M. El-Dahr, Patricia C. Chulada, Mosanda M. Mvula, L. Faye Grimsley, Maureen Y. Lichtveld, LuAnn E. White, Yvonne M. Sterling, Kevin U. Stephens, William J. Martin

**Affiliations:** 1 Rho Federal Systems Division, Inc., Chapel Hill, North Carolina, USA; 2 SRA International, Inc., Durham, North Carolina, USA; 3 Visionary Consulting Partners, LLC, Fairfax Station, Virginia, USA; 4 Department of Pediatrics, Tulane University School of Medicine, New Orleans, Louisiana, USA; 5 Clinical Research Program, National Institute of Environmental Health Sciences, National Institutes of Health, Department of Health and Human Services, Research Triangle Park, North Carolina, USA; 6 New Orleans Health Department, New Orleans, Louisiana, USA; 7 School of Public Health and Tropical Medicine, Tulane University, New Orleans, Louisiana, USA; 8 Health Sciences Center School of Nursing, Louisiana State University, New Orleans, Louisiana, USA; 9 National Institute of Child Health and Development, National Institutes of Health, Department of Health and Human Services, Bethesda, Maryland, USA

**Keywords:** asthma case management, asthma counselor, asthma morbidity, environmental intervention, Hurricane Katrina, indoor allergens, mold

## Abstract

Background: Childhood asthma morbidity and mortality in New Orleans, Louisiana, is among the highest in the nation. In August 2005, Hurricane Katrina created an environmental disaster that led to high levels of mold and other allergens and disrupted health care for children with asthma.

Objectives: We implemented a unique hybrid asthma counselor and environmental intervention based on successful National Institutes of Health asthma interventions from the National Cooperative Inner City Asthma (NCICAS) and Inner-City Asthma (ICAS) Studies with the goal of reducing asthma symptoms in New Orleans children after Hurricane Katrina.

Methods: Children (4–12 years old) with moderate-to-severe asthma (*n* = 182) received asthma counseling and environmental intervention for approximately 1 year. HEAL was evaluated employing several analytical approaches including a pre–post evaluation of symptom changes over the entire year, an analysis of symptoms according to the timing of asthma counselor contact, and a comparison to previous evidence-based interventions.

Results: Asthma symptoms during the previous 2 weeks decreased from 6.5 days at enrollment to 3.6 days at the 12-month symptom assessment (a 45% reduction, *p* < 0.001), consistent with changes observed after NCICAS and ICAS interventions (35% and 62% reductions in symptom days, respectively). Children whose families had contact with a HEAL asthma counselor by 6 months showed a 4.09-day decrease [95% confidence interval (CI): 3.25 to 4.94-day decrease] in symptom days, compared with a 1.79-day decrease (95% CI: 0.90, 2.67) among those who had not yet seen an asthma counselor (*p* < 0.001).

Conclusions: The novel combination of evidence-based asthma interventions was associated with improved asthma symptoms among children in post-Katrina New Orleans. Post-intervention changes in symptoms were consistent with previous randomized trials of NCICAS and ICAS interventions.

Asthma affects an estimated 9.1% of U.S. children < 18 years of age (Akinbami et al. 2009). Disproportionately high levels of asthma morbidity and mortality are seen in low-socioeconomic and minority populations (Akinbami et al. 2011), with an asthma mortality rate seven times higher among black children than white children (Akinbami et al. 2009). Although little is known about the factors that lead to the development of asthma, researchers have identified a broad range of triggers that are associated with asthma symptoms and exacerbation. These factors include exposure to high levels of indoor allergens, irritants, and mold (Gruchalla et al. 2005; Kattan et al. 2007; Perzanowski and Platts-Mills 2009; Pongracic et al. 2010) and high levels of indoor and outdoor pollutants (Kattan et al. 2007; McConnell et al. 1999; O’Connor et al. 2008). Barriers to quality health care and poor continuity of health care are associated with increased asthma morbidity and are especially common in lower-income urban areas (Crain et al. 1998; Crocker et al. 2009). Evidence-based interventions that support the reduction of environmental exposures and facilitate access to care and asthma self-management may address these issues in vulnerable populations.

Even before Hurricane Katrina, Louisiana and the City of New Orleans and surrounding parishes (NOLA) had some of the highest rates of asthma prevalence in the nation (Akinbami et al. 2009; Mvula et al. 2005). Given the environmental impact of Hurricane Katrina in August 2005, one might expect that children with asthma would be at increased risk. To address the multidimensional nature of the problems in post-Katrina NOLA, the National Institute of Environmental Health Sciences (Research Triangle Park, NC), the National Center on Minority Health and Health Disparities (Bethesda, MD), and the Merck Childhood Asthma Network, Inc. (Washington, DC), came together under the auspices of the Foundation for the National Institutes of Health (NIH; Bethesda, MD) to support an asthma initiative in post-Katrina NOLA.

This initiative, Head-off Environmental Asthma in Louisiana (HEAL), sought to reduce asthma morbidity in children with asthma by employing a combination of evidence-based interventions shown to be efficacious in randomized, controlled clinical trials (Evans et al. 1999; Morgan et al. 2004). These interventions were developed over the past decade as part of the National Cooperative Inner City Asthma (NCICAS) and Inner-City Asthma (ICAS) Studies. These two studies provided patient-tailored counseling in the clinic and home to supply the caretakers of children with asthma with knowledge, skills, and motivation to manage their childen’s asthma and reduce exposures to allergens in the home environment. HEAL adapted components of these two evidence-based interventions into a novel, field-applicable, hybrid asthma counselor and environmental intervention to provide support for families with asthma in the aftermath of Hurricane Katrina. In this report we present the results of our effort to translate knowledge from randomized clinical trials to a real-world setting amid unprecedented challenges resulting from a major natural disaster.

## Methods

*Study design and population.* HEAL was an observational, pre–post intervention study that recruited NOLA children 4–12 years of age with moderate-to-severe persistent asthma, as defined by the National Asthma Education and Prevention Program (National Heart, Lung, and Blood Institute 2007), over 1 year. The major goal was to determine whether a novel combination of the evidence-based NCICAS asthma counselor and ICAS environmental interventions could be effective in a post-disaster setting (Evans et al. 1999; Morgan et al. 2004). Another goal of HEAL (not covered in this report) was to characterize relationships between environmental exposures and childhood asthma morbidity.

Details regarding the study design, eligibility requirements, recruitment methods, and characteristics of the HEAL study population are provided in the accompanying article (Chulada et al. 2012). In brief, HEAL was initially designed as a randomized, interventional trial in which 225 children would receive an excellent standard of care, and another 225 would receive the same excellent standard of care plus the intervention. Due to a low recruitment rate, HEAL was transitioned into an observational design in which all enrolled children became eligible for the intervention, including those already randomized to the control group. Although randomized control and intervention groups could not be compared, we were able to compare asthma symptoms among children who saw an asthma counselor earlier in the study versus those who had not. HEAL was approved by the NIEHS, Tulane University, and Louisiana State University institutional review boards; caretakers were asked for written informed consent, and children were asked for written (≥ 7 years of age) or oral (< 7 years of age) assent, depending on age.

*Intervention.* The HEAL asthma counselors worked in teams with community health workers. The asthma counselors had master’s degrees in health-related fields, public health backgrounds, and experience with counseling and community outreach. All had prior experience working with individuals with a chronic disease. The community health workers worked closely with the asthma counselors and helped with several aspects of the study, including building rapport with the caretakers and scheduling visits.

The HEAL intervention was modeled after the intervention used in NCICAS (Evans et al. 1999) in which the asthma counselor helped the caretaker to communicate with the child’s school nurse, primary care provider, and others involved in caring for the child to develop and implement an asthma action plan individualized to the child. The intervention was tailored to each participant’s allergen sensitivities and environmental exposures. In addition, participant-tailored interventions addressed potential problems related to adherence to medication use as well as potential problems with insurance, access to care, and health care attitudes, as identified by risk assessment tools (Crain et al. 2002; Evans et al. 1999) and prioritized by the asthma counselor. The HEAL intervention also included a participant-tailored environmental component modeled after ICAS (Morgan et al. 2004). Specifically, the HEAL asthma counselor guided the caretaker in the use of materials provided in an environmental kit (e.g., HEPA air cleaner, wet and dry mops, mattress and pillow encasements, cleaning supplies, food storage containers). The goal of the environmental component was to provide the caretaker with the knowledge, skills, motivation, and supplies to reduce the child’s exposure to the allergens to which they were sensitive.

At least two in-person asthma counselor visits were attempted with each caretaker during the intervention year, one in a study office located in a community facility (such as a clinic, library, or school), and the second at the caretaker’s home. The asthma counselor followed each visit with a phone call within 2 weeks.

*Study visits.* At baseline, participants completed a clinical evaluation, including an assessment of symptoms, blood collection, and allergen skin testing (Chulada et al. 2012). In addition, trained clinic staff conducted interviews and filled out the study questionnaires during baseline clinic visits. A baseline home evaluation was conducted 1–27 weeks (median, 3 weeks) after the baseline clinical evaluation; repeat baseline home evaluations were conducted for 14 participants who moved in the first 11 months of the study. Briefly, this evaluation included a visual inspection of the home, air and dust sample collection for mold and allergens, and measurements of indoor air temperature, humidity, and moisture, as described in detail in the accompanying article (Grimsley et al. 2012). Upon completion of both the baseline clinical and home evaluations, children were assigned an asthma counselor. Participants in the control group under the original study design were assigned an asthma counselor when the study was changed from randomized to observational. Clinical evaluations were repeated at month 12, and additional home evaluations were targeted for months 6 and 12. Trained nursing personnel interviewed caretakers by telephone 3, 6, 9, and 12 months after enrollment to obtain information on symptoms, health care utilization, and medication use.

*Statistical methods*. Consistent with the NCICAS and ICAS studies, the primary outcome in HEAL was the maximum symptom days (MSDs) in the previous 2 weeks, which was the largest value among three asthma symptom variables: *a*) the number of days with wheezing, tightness in the chest, or cough; *b*) the number of nights with disturbed sleep as a result of asthma; and *c*) the number of days the child had to slow down or discontinue play activities because of asthma. Asthma symptoms were assessed up to five times during the HEAL project (at baseline and at 3, 6, 9, and 12 months).

Four approaches were taken to evaluate the impact of the intervention. First, we compared mean symptom levels at baseline and after 12 months in the HEAL study population to means from the ICAS and NCICAS intervention groups. Baseline to 12-month changes were tested for each study using *t*-tests. Second, we compared the change in MSDs for HEAL participants based on the timing of the first contact with an asthma counselor using a *t*-test. Third, we conducted an ANCOVA (analysis of covariance) with adjustment for baseline MSD levels comparing MSDs among children who saw an asthma counselor by either 6 or 12 months with symptoms among children who did not, regardless of their randomization status. We conducted similar analyses for several secondary outcomes in HEAL (medication usage and airborne mold, IgE, and allergen levels). For context, we also calculated intervention effects for MSDs at comparable time points in ICAS and NCICAS, which are adjusted for site along with baseline MSD, to improve consistency with the previous manuscripts. Fourth, we investigated differences in the results in HEAL according to baseline characteristics, such as mold exposure, atopy, and IgE, using mixed models with interaction terms between intervention status (based on timing of asthma counselor contact) and each baseline characteristic. Models were adjusted for baseline MSD levels.

Six-month results are presented along with 12-month results when possible, and analyses showing full results at 6 months are included as supplemental tables [see Supplemental Material (http://dx.doi.org/10.1289/ehp.1104242)]. Participants with missing data are excluded, resulting in 153 and 159 participants in the 6- and 12-month analyses, respectively; as a result, baseline values may differ slightly from previous reports using the full 182-participant study population at baseline.

## Results

Baseline demographic and environmental characteristics of the 182 HEAL children have been described in detail elsewhere (Chulada et al. 2012; Grimsley et al. 2012). In short, HEAL children were mostly African American (67%), and 51% were from households making < $30,000 per year. Most HEAL participants (62%) had water damage to their homes as a result of Katrina, with 24% reporting flooding, 25% reporting roof damage, and 14% reporting both flooding and roof damage, compared with 38% who reported no damage. HEAL children had an average of 1.9 in-person meetings with asthma counselors over the course of the study (range, 0–4 visits), with 123 of 182 (68%) children having two or more contacts (in-person visits and asthma counselor telephone calls).

**Figure 1:**
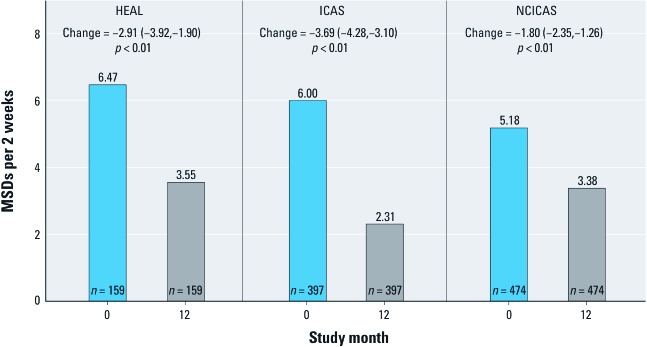
MSDs during the previous 2 weeks at baseline (blue) and 12 months (gray) for HEAL (all participants), ICAS, and NCICAS participants who received the study intervention. Changes in MSDs are shown with 95% CIs and p-values comparing symptoms at baseline and 12 months.

Children in HEAL showed a marked decrease in symptoms over the course of the study, from 6.47 days during the 2 weeks before enrollment to 3.55 days at the 12-month symptom assessment [*n* = 159 participants who completed 12-month symptom phone call, –2.92 days; 95% confidence interval (CI): –3.92, –1.26, representing a 45% reduction, *p* < 0.001]. The magnitude of the change was comparable to changes observed in the MSDs in the ICAS and NCICAS intervention groups, which had reductions of 62% (from 6.00 to 2.31 MSDs, or –3.69 days; 95% CI: –4.28, –3.10) and 35% (5.18 to 3.38 MSDs, or –1.80 days; 95% CI: –2.35, –1.26), respectively (both *p* < 0.001) (Figure 1).

**Figure 2:**
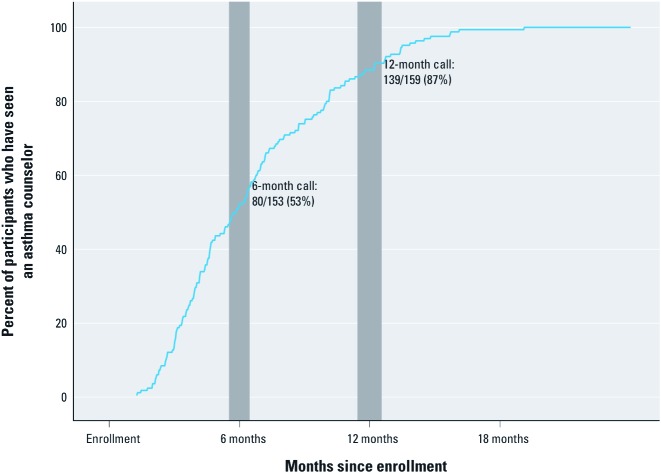
The timings of the participants’ first contacts with an asthma counselor in HEAL are shown. Although all HEAL participants were eligible for the intervention, some did not see an asthma counselor until later in the study. Values exclude participants who missed the given assessment (month 6: 29/182; month 12: 23/182). Shaded gray bars represent the interquartile range for the timing of the 6- and 12-month symptom assessment excluding participants who missed the visits.

Of the 153 HEAL children who had a 6-month symptom assessment call, 80 (53%) had seen an asthma counselor before the call (Figure 2). By the 12-month call, 87% of the children had met with their asthma counselor (139 of 159). Baseline asthma symptoms (i.e., MSDs, pulmonary function, and controller medication) were similar between children who had seen a counselor before the 12-month assessment and those who had not (*n* = 20), but there were significant differences between the groups in demographic characteristics (63% vs. 95% from Orleans Parish, 63% vs. 85% African American), environmental exposures (3,281 vs. 8,408 outdoor mold spores/m^3^), and season of enrollment (36% vs. 95% enrolled in the spring or summer of 2007) (Table 1). Similar patterns of differences in baseline characteristics were observed between children who had asthma counselor contact before versus after 6 months [see Supplemental Material, Table S1 (http://dx.doi.org/10.1289/ehp.1104242)].

**Table 1:** Baseline characteristics by timing of the first contact with an asthma counselor.

Characteristic	Contact with asthma counselor before 12 months			*p*-Value
	Yes (*n* = 139)	No (*n* = 20)		
Symptoms	6.6 ± 5.0	5.8 ± 3.9	0.48
Parish	0.004
Orleans	88/139 (63%)	19/20 (95%)
Jeffersona	51/139 (37%)	1/20 (5%)
Enrollment season	< 0.001
Spring 2007	14/139 (10%)	15/20 (75%)
Summer 2007	36/139 (26%)	4/20 (20%)
Fall 2007	49/139 (35%)	—
Winter 2007	32/139 (23%)	1/20 (5%)
Spring 2008	8/139 (6%)	—
Race/ethnicity	0.03
African American	88/139 (63%)	17/20 (85%)
Hispanic	9/139 (6%)	2/20 (10%)
Other	42/139 (30%)	1/20 (5%)
Income < $15,000	30/128 (23%)	7/19 (37%)	0.26
Female sex	61/139 (44%)	12/20 (60%)	0.81
Lung function
FEV1% predicted	90.7 ± 17.7	98.3 ± 15.1	0.25
FEV1/FVC	77.8 ± 10.5	80.0 ± 8.3	0.57
Taking Inhaled corticosteroids	81/139 (58%)	11/20 (55%)	0.81
Mold levels (spores/m3)
Outdoor total	3,281 (445)	8,408 (2,299)	0.01
Indoor total	480 (52)	675 (237)	0.28
IgE (serum)
Total IgE (kU/L)	130 (38)	240 (32)	0.10
Positive mold-specific IgE	74/133 (56%)	7/19 (37%)	0.63
Exposure to allergens in dust
Alternaria > 10 µg/g	86/138 (62%)	7/20 (35%)	0.03
Detectable roach	30/138 (22%)	3/20 (15%)	0.77
Detectable dust mite	54/138 (39%)	5/20 (25%)	0.32
Detectable mouse	84/138 (61%)	12/20 (60%)	0.99
Skin test result
Alternaria	72/138 (52%)	11/20 (55%)	0.99
Roach	69/138 (50%)	12/20 (60%)	0.48
Dust mite	89/138 (64%)	16/20 (80%)	0.21
Mouse	39/138 (28%)	5/20 (25%)	0.99
Abbreviations: FEV1, forced expiratory volume in 1 sec; FVC, forced vital capacity. Values are n (%), mean ± SD, or geometric mean (geometric SD). aAlso includes St.Tammany and St. Bernard.						

**Figure 3:**
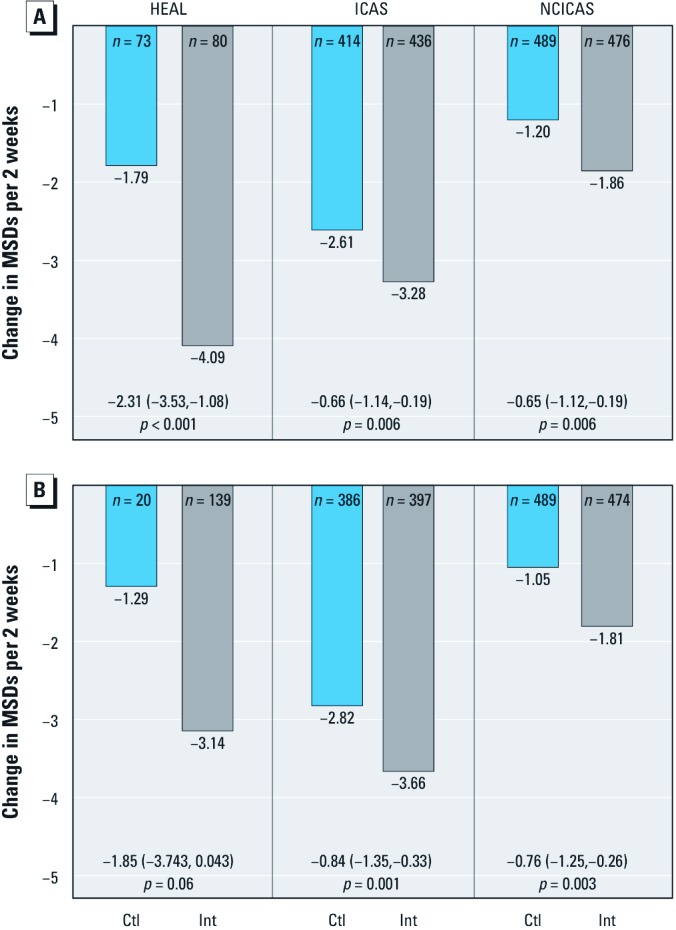
Change in MSDs between baseline and the 6-month assessment (*A*) and between baseline and the 12-month assessment (*B*) in HEAL, ICAS, and NCICAS. ICAS and NCICAS participants are grouped by their treatment intervention (Int) or control (Ctl) randomization assignment, and HEAL participants are grouped by whether they had contact with an asthma counselor before the given assessment (Ctl: no intervention before assessment; Int: received intervention before assessment). Values are effect sizes (95% CIs) and p-values. ICAS and NCICAS estimates are adjusted for site.

At the 6-month symptom phone call, the 80 children who had seen an asthma counselor had a 4.09-MSD reduction from baseline, compared with a 1.79-day decrease for the 73 children who had not yet seen an asthma counselor (–2.31 days; 95% CI: –3.53, –1.08; *p* < 0.001). At 12 months, the group that had seen a counselor had a 3.14-day improvement compared with a 1.29-day improvement for those who had not (–1.85 days; 95% CI: –3.74, 0.04; *p* = 0.06). The effect sizes seen in ICAS and NCICAS were somewhat smaller but showed greater statistical significance because the studies included more children and had a more powerful study design (i.e., randomized, controlled trial) than did HEAL. In ICAS, there was a 0.66-day intervention effect at 6 months and a 0.84-day effect at 1 year (both *p* < 0.01); similar effects were seen in NCICAS (0.65- and 0.76-day effects at 6 and 12 months, respectively, both *p* < 0.01) (Figure 3).

Children who saw an asthma counselor in the first 6 months of the study had decreased symptoms relative to baseline (4.14 days decrease at month 6 among the 77 participants who completed all symptom assessments). These same children had significantly less asthma counselor contact in the second half of the study (0.6 average contacts compared with 1.6 in the first 6 months, *p <* 0.001), with a corresponding partial rebound for symptom level (1.17-day increase from 6 to 12 months, *p* = 0.07, Table 2). Children who saw an asthma counselor for the first time after 6 months of the study also showed a statistically significant decrease in symptoms in the first 6 months (1.88-day decrease for 52 participants, *p* = 0.04); however, this decrease in symptoms was more modest when compared with participants who had already seen an asthma counselor in the first 6 months (*p* = 0.02). Also, the children who did not see an asthma counselor until after the first 6 months saw their counselors an average of 1.8 times each during the second 6 months and saw a corresponding larger symptom improvement (1.13-day decrease vs. 1.17-day increase for participants who first saw an asthma counselor in the first 6 months, *p* = 0.01).

**Table 2:** Changes in MSDs during the previous 2 weeks by timing of the first contact with an asthma counselor.

First asthma counselor contact	n^a^	Symptoms by month					Change in symptoms						
							Change (95% CI) 0→6^b^	Change (95% CI) 6→12^b^
		Baseline	6	12	*p*-diff^c^	*p*-diff^c^		
Before 6 months	77	6.52	2.38	3.55	–4.14 (–5.50, –2.79)d	0.02	1.17 (–0.10, 2.43)	0.01
After 6 months	52	5.83	3.94	2.81	–1.88 (–3.71, –0.06)	–1.13 (–2.67, 0.40)d
aThe 41 participants with missing symptom assessments and 20 participants who never saw an asthma counselor are excluded. bChange in MSD between symptom assessments at baseline and 6 months (Change 0→6) and 6 months and 12 months (Change 6→12) with 95% CIs. cTests for a difference (p-diff) in change over time by timing of the first asthma counseling contact. dMeasures corresponding to the first asthma counselor contact.																

Levels of mold exposure in HEAL showed marked decreases between the baseline and 12-month home evaluations. The bedroom airborne mold exposure dropped from 522 to 266 spores/m^3^, and the *Alternaria* levels in bedroom dust dropped from 11.6 to 6.0 μg/g (both *p* < 0.001). However, it is not possible to determine if the intervention played a role in these decreases. Although children who saw an asthma counselor before 12 months had lower levels of IgE, dust-based allergen exposure, and indoor airborne mold exposure at 12 months than children who had not seen a counselor, data were available for only 15 children in the latter group, and none of the differences reached statistical significance (all *p* > 0.25) (Table 3). Participants who had seen an asthma counselor in the first 6 months also showed nominally improved environmental conditions for most outcomes (airborne mold; dust-based roach, mite, and *Alternaria*) at the 6-month home evaluation visit when compared with those who had not seen an asthma counselor in that time frame [all *p* > 0.15; see also Supplemental Material, Table S2 (http://dx.doi.org/10.1289/ehp.1104242)]. The only exception was the level of mouse allergen, which was 18 percentage points higher for the participants with asthma counselor contact (*p* = 0.06).

**Table 3:** Symptoms at 12 months, exposures, and allergic characteristics by timing of the first contact with an asthma counselor.

Contact with asthma counselor before 12 months							*p*-Value
Yes			No				
*n*	Mean ± SE	*n*	Mean ± SE	Difference			
Maximum symptom days	139	3.32 ± 0.3	20	5.17 ± 0.9	–1.85	0.05
Taking medication (%)	121	68.3 ± 4.6	16	43.7 ± 13.6	24.6	0.09
Mold levels (spores/m3)
Outdoor total	131	2,576 ± 462.5	15	1,364 ± 731.8	1,212	0.26
Indoor total	131	258 ± 27.4	15	356 ± 112.1	–98	0.34
IgE (serum)
Total (kU/L)	111	274 ± 16.6	15	284 ± 47.4	–10	0.84
Detectable mold IgE (%)	112	44.4 ± 11.5	15	62.7 ± 24.3	–18.3	0.51
Allergens in dust (%)
Alternaria > 10 µg/g	126	29.9 ± 4.2	15	44.3 ± 13.4	–14.4	0.29
Detectable roach	126	31.9 ± 4.2	15	27.8 ± 11.9	4.1	0.76
Detectable dust mite	126	35.6 ± 4.4	15	49.3 ± 13.3	–13.7	0.32
Detectable mouse	126	53.3 ± 4.6	15	67.9 ± 12.4	–14.6	0.31
Values in each row are adjusted for baseline levels of the given outcome.												

In addition to reductions in asthma symptoms, children who saw an asthma counselor before 12 months were also more likely to be taking inhaled corticosteroids (ICS) at the end of the study (68.3% compared with 43.7%, *p* = 0.09) (Table 3). We found that associations between asthma counselor contact and MSD were generally the same regardless of baseline participant characteristics. Although participants taking ICS at baseline did see larger MSD decreases if they had asthma counselor contact at 12 months (3.51-day decrease vs. 0.20-day increase for those not taking ICS, *p* = 0.055), sample size was generally inadequate for this type of comparison and other baseline characteristics (airborne mold, IgE, and dust allergen levels) showed no relationship (Table 4). The 6-month findings were similar, with only parish being a significant predictor of increased response by 6 months [3.00-day decrease in Orleans compared with 0.21-day decrease in other parishes, *p* = 0.04; see also Supplemental Material, Table S3 (http://dx.doi.org/10.1289/ehp.1104242)].

**Table 4:** Differences in MSDs according to intervention status at 12 months and baseline characteristics.

Baseline status	Contact with asthma counselor before 12 months						
	No			Yes			Difference			Interaction *p*-Value
	*n*	Mean	*n*	Mean	Mean	*p*-Value				
Parish
Jefferson	1	5.08	51	3.12	–1.97	0.630	0.96
Orleans	19	5.18	88	3.44	–1.74	0.089
Taking inhaled corticosteroids
No	9	3.20	58	3.40	0.20	0.890	0.05
Yes	11	6.78	81	3.27	–3.51	0.007
Outdoor mold
< 1,000 spores/m3	1	5.45	31	3.57	–1.88	0.647	0.99
≥ 1,000 spores/m3	19	5.16	108	3.25	–1.91	0.059
Bedroom mold
< 1,000 spores/m3	11	5.78	105	3.26	–2.52	0.049	0.42
≥ 1,000 spores/m3	9	4.43	34	3.51	–0.92	0.544
Total IgE
< 100 kU/L	5	7.61	36	3.29	–4.32	0.026	0.17
≥ 100 kU/L	14	4.60	95	3.37	–1.23	0.285
Detectable mold IgE
No	12	6.36	74	3.38	–2.98	0.018	0.22
Yes	7	3.70	59	3.23	–0.46	0.771
Alternaria
< 10 µg/g	13	5.92	52	3.56	–2.36	0.060	0.38
≥ 10 µg/g	7	3.80	86	3.21	–0.59	0.710
Detectable roach
No	17	5.60	108	3.41	–2.19	0.039	0.35
Yes	3	2.82	30	3.11	0.29	0.906
Detectable dust mite
Yes	15	5.50	84	3.37	–2.13	0.062	0.58
No	5	4.20	54	3.30	–0.90	0.638
Detectable mouse
Yes	8	5.69	54	2.54	–3.14	0.039	0.27
No	12	4.83	84	3.86	–0.97	0.431

## Discussion

Translating evidence-based interventions to real-world settings is difficult under the best of circumstances. We had anticipated that this would be an extraordinary challenge in the aftermath of Hurricane Katrina, one of the United States’ most devastating disasters. However, given the substantial needs of the families and children with asthma in this community, which already had one of the highest asthma rates in the country, we felt this effort was critically important.

This study demonstrates that evidence-based asthma counselor and environmental interventions to improve asthma management and reduce environmental exposures can be combined and effectively implemented in a post-disaster setting. The application of this novel combination of the ICAS and NCICAS interventions was associated with a decrease in asthma symptoms that was consistent with differences observed in intervention versus non-intervention groups in these earlier, tightly controlled NIH studies.

In the NCICAS intervention, asthma counselors helped the caretakers to manage their child’s asthma and emphasized the importance of proper medication use and avoidance of environmental triggers, although no home visits or direct demonstrations of environmental risk factor mitigation were undertaken (Evans et al. 1999). The asthma counselors in NCICAS began their interventions with two group asthma counseling sessions, followed by in-clinic visits every other month and telephone calls on the alternate months over the course of a year, for a total of 6 clinic visits and 6 calls. The ICAS environmental intervention did not provide asthma counseling nor did it involve the primary care physicians, but rather had environmental mitigation staff provided remediation of identified environmental risks (based on the child’s sensitivity and exposure) and discussed ways to change behavior to reduce or eliminate those risks during the course of 4–7 home visits for each family (Morgan et al. 2004).

These NIH-funded interventions (NCICAS and ICAS) were quite different in nature, and both were effective but very demanding on the staff and the families. In consideration of the many demands and difficulties being encountered by the post-Katrina NOLA population, the maximum number of contacts with HEAL participants was reduced to two in-person visits (one of which took place in the home) and up to two follow-up telephone contacts. However, even though considerably fewer home visits than ICAS and fewer asthma counselor contacts than NCICAS occurred in HEAL, HEAL appeared to be similarly effective. This outcome suggests that the use of asthma counselors who are well trained in asthma counseling and home environmental interventions is applicable to the community setting and that similar strategies might be useful in other resource-limited or post-disaster settings to improve the health of children with respiratory and other health conditions.

In the uncontrolled settings of translational and observational studies, limitations must be considered when interpreting the findings. There were differences in demographics, environment, and season of enrollment between those who saw an asthma counselor before the 12-month symptom assessment and those who did not. Many of these differences between the groups were a result of limitations imposed by the recruitment constraints that led to modifications in the study design. Children randomized to the non-intervention group before the study redesign and before the recruitment area had been expanded beyond Orleans Parish were more highly represented in the group who had not seen an asthma counselor by 6 or 12 months. In fact, 19 of the 20 children who did not see an asthma counselor before their 12-month symptom call were from Orleans Parish. There were differences between HEAL participants from Orleans Parish versus those from other parishes, which are described elsewhere (Chulada et al. 2012), and these differences may have influenced associations between the intervention and symptoms in HEAL. Although the intervention showed a trend toward reduced allergen levels, concomitant changes were occurring following Katrina; a natural reduction of mold levels and home renovations and remediation also took place after the storm (Grimsley et al. 2012) and undoubtedly contributed to improvements in asthma symptoms.

The Inner-City Asthma Intervention, which was an earlier implementation of the asthma counselor NCICAS intervention in community settings conducted by the Centers for Disease Control and Prevention, reported challenges similar to some faced in HEAL (Love and Spiegel 2006; Williams and Redd 2006). In general, there are many considerations when translating clinical trials into clinical practice, which have been well described (Glasgow and Emmons 2007; Green et al. 2009). Although the HEAL project faced many of these problems as well as the additional challenges presented by the post-Katrina setting, we believe this effort demonstrated the value and effectiveness of translating evidence-based clinical trials into real world settings, even in a community struggling to recover from the most costly and destructive disaster to ever occur in the United States.

## Supplemental Material

(397 KB) PDF
