# Supplementary material for: Implementation of Evidence-based Asthma Interventions in Post-Katrina New Orleans: The Head-off Environmental Asthma in Louisiana (HEAL) Study
Source: Environ Health Perspect. 2012 Aug 15;120(11):1607–12. doi: 10.1289/ehp.1104242 (PMC3556603; doi:10.1289/ehp.1104242)
Supplement: (397 KB) PDF [file ehp.1104242.s001.pdf]

Supplemental Material

Implementation of Evidence-based Asthma Interventions in Post-Katrina New Orleans: The Head-off Environmental Asthma in Louisiana (HEAL) Study

Herman Mitchell, Richard D. Cohn, Jeremy Wildfire, Eleanor Thornton, Suzanne Kennedy, Jane M. El-Dahr, Patricia C. Chulada, Mosanda M. Mvula, L. Faye Grimsley, Maureen Y. Lichtveld, LuAnn E. White, Yvonne M. Sterling, Kevin U. Stephens, Sr., William J. Martin II

Contents

Supplemental Material, Table S1. Baseline characteristics by timing of the first contact with an asthma counselor 3

Supplemental Material, Table S2. Symptoms at 6 months, exposures, and allergic characteristics by timing of the first contact with an asthma counselor 4

Supplemental Material, Table S3. Differences in Maximum Symptom Days (MSD) according to intervention status at 6 months and baseline characteristics........ 5

Supplemental Table 1 – Baseline characteristics by timing of the first contact with an asthma counselor

	Contact with asthma counselor Before 6 Months		*p*
	**Yes (N=80)**	**No (N=73)**	
Symptoms	6.6 ± 5.0	6.0 ± 4.7	0.44
Parish			0.07
Orleans	48/80 (60%)	54/73 (74%)	
Jefferson	32/80 (40%)	19/73 (26%)	
Enrollment Season			<0.001
Spring 2007	5/80 (6%)	11/73 (15%)	
Summer 2007	13/80 (16%)	31/73 (42%)	
Fall 2007	26/80 (33%)	24/73 (33%)	
Winter 2007	29/80 (36%)	4/73 (6%)	
Spring 2008	7/80 (9%)	3/73 (4%)	
Race/ethnicity			0.33
African American	49/80 (61%)	51/73 (70%)	
Hispanic	5/80 (6%)	6/73 (8%)	
Other	26/80 (33%)	16/73 (22%)	
Income <$15,000	18/74 (24%)	17/68 (25%)	0.93
Gender - Female	34/80 (43%)	36/73 (49%)	0.40
Lung function			
FEV1 % predicted	91.2 ± 86.0	90.2 ± 83.9	0.81
FEV1/FVC	78.0 ± 11.2	78.8 ± 8.7	0.74
Taking Inhaled Corticosteroids	46/80 (58%)	41/73 (56%)	0.87
Mold levels (spores/m^3)			
Outdoor total	2322 (378)	6475 (1235)	<0.001
Indoor total	388 (45)	618 (105)	0.03
IgE (serum)			
Total IgE (kU/L)	208 (37)	207 (36)	0.99
Positive Mold-specific IgE	34/77 (44%)	31/69 (45%)	0.93
Allergens in dust			
Alternaria >10 µg/g	52/80 (65%)	42/72 (58%)	0.40
Detectable roach	14/80 (18%)	17/72 (24%)	0.35
Detectable dust mite	29/80 (36%)	24/72 (33%)	0.71
Detectable mouse	45/80 (56%)	47/72 (65%)	0.26
Skin Test Result			
Alternaria	42/80 (53%)	39/71 (55%)	0.77
Roach	411/80 (51%)	33/71 (46%)	0.56
Dust Mite	50/80 (63%)	50/71 (70%)	0.30
Mouse	20/80 (25%)	19/71 (27%)	0.81

Values are counts (percentages), mean ± standard deviation, or geometric mean (geometric standard deviation).

Supplemental Table 2 –Symptoms at 6 months, exposures and allergic characteristics by timing of the first contact with an asthma counselor

	Contact with Asthma Counselor Before 6 Months					
		Yes		No		
	N	Mean (SE)	N	Mean (SE)	Difference	*p*
Maximum Symptom Days	80	2.25 (0.4)	73	4.56 (0.5)	-2.31	<0.001
Mold levels (spores/m^3)						
Outdoor total	86	2166 (365.2)	49	1818 (412.0)	-348	0.55
Indoor total	87	456 (97.4)	49	320 (97.4)	-136	
Allergens in dust						
Alternaria >10 µg/g	83	26.7% (22.2)	47	66.3% (30.8)	-39.6%	0.30
Detectable Roach	83	44.5% (5.5)	47	31.9% (6.8)	-12.6%	0.16
Detectable Dust Mite	83	39.7% (5.5)	47	28.1% (6.7)	-11.6%	0.20
Detectable Mouse	83	47.5% (5.8)	47	65.8% (7.3)	+18.3%	0.06

Values in each row are adjusted for baseline levels of the given outcome.

Supplemental Table 3 – Differences in Maximum Symptom Days (MSD) according to intervention status at 6 months and baseline characteristics

	Contact with Asthma Counselor Before 6 Months						
	No		Yes		Difference		Interaction
**Baseline Status**	**N**	**Mean**	**N**	**Mean**	**Mean**	***p***	***p***
Parish							
Jefferson	19	2.26	32	2.05	-0.21 (-2.4, 1.9)	0.84	0.04
Orleans	54	5.38	48	2.38	-3.00 (-4.5, -1.5)	<0.0001	
Taking Inhaled Corticosteroids							
No	32	3.44	34	1.70	-1.74 (-3.6, 0.1)	0.06	0.41
Yes	41	5.43	46	2.66	-2.76 (-4.4, -1.2)	<0.001	
Outdoor Mold							
<1000 spores/m^3	4	7.90	26	2.61	-5.26 (-9.3, -1.2)	0.01	0.17
≥1000 spores/m^3	69	4.36	54	2.08	-2.28 (-3.7, -0.9)	0.001	
Bedroom Mold							
<1000 spores/m^3	51	4.29	66	2.26	-2.03 (-3.5, -0.6)	0.005	0.53
≥1000 spores/m^3	22	5.18	14	2.20	-2.98 (-5.6, -0.3)	0.03	
Total IgE							
<100 KU/L	18	4.64	24	2.49	-2.15 (-4.6, 0.3)	0.08	0.88
≥100 KU/L	51	4.53	51	2.16	-2.37 (-3.9, -0.8)	0.003	
Detectable Mold IgE							
No	38	5.27	43	2.22	-3.06 (-4.8, -1.4)	<0.001	0.19
Yes	31	3.66	34	2.32	-1.34 (-3.2, 0.6)	0.17	
Alternaria							
<10 µg/g	30	4.62	28	2.52	-2.11 (-4.1, -0.1)	0.04	0.85
≥10 µg/g	42	4.47	52	2.12	-2.35 (-3.9, -0.8)	0.004	
Detectable roach							
No	55	4.49	66	2.09	-2.40 (-3.8, -1.0)	<0.001	0.61
Yes	17	4.67	14	3.06	-1.61 (-4.4, 1.2)	0.25	
Detectable dust mite							
No	48	4.62	51	2.76	-1.86 (-3.4, -0.3)	0.02	0.39
Yes	24	4.36	29	1.37	-2.99 (-5.1, -0.9)	0.006	
Detectable mouse							
No	25	3.55	35	2.22	-1.34 (-3.3, 0.6)	0.19	0.27
Yes	47	5.05	45	2.29	-2.76 (-4.3, -1.2)	<0.001	
